# Bayesian semi-supervised classification of bacterial samples using MLST databases

**DOI:** 10.1186/1471-2105-12-302

**Published:** 2011-07-26

**Authors:** Lu Cheng, Thomas R Connor, David M Aanensen, Brian G Spratt, Jukka Corander

**Affiliations:** 1 Department of Mathematics and statistics, P.O.Box 68, University of Helsinki, 00014, Finland; 2 Department of Infectious Disease Epidemiology, Imperial College London, Norfolk Place, London W2 1PG, UK; 3 Sanger Institute, Wellcome Trust Genome Campus, Hinxton, Cambridge, CB10 1SA, UK

## Abstract

**Background:**

Worldwide effort on sampling and characterization of molecular variation within a large number of human and animal pathogens has lead to the emergence of multi-locus sequence typing (MLST) databases as an important tool for studying the epidemiology and evolution of pathogens. Many of these databases are currently harboring several thousands of multi-locus DNA sequence types (STs) enriched with metadata over traits such as serotype, antibiotic resistance, host organism etc of the isolates. Curators of the databases have thus the possibility of dividing the pathogen populations into subsets representing different evolutionary lineages, geographically associated groups, or other subpopulations, which are defined in terms of molecular similarities and dissimilarities residing within a database. When combined with the existing metadata, such subsets may provide invaluable information for assessing the position of a new set of isolates in relation to the whole pathogen population.

**Results:**

To enable users of MLST schemes to query the databases with sets of new bacterial isolates and to automatically analyze their relation to existing curated sequences, we introduce here a Bayesian model-based method for semi-supervised classification of MLST data. Our method can use an MLST database as a training set and assign simultaneously any set of query sequences into the earlier discovered lineages/populations, while also allowing some or all of these sequences to form previously undiscovered genetically distinct groups. This tool provides probabilistic quantification of the classification uncertainty and is highly efficient computationally, thus enabling rapid analyses of large databases and sets of query sequences. The latter feature is a necessary prerequisite for an automated access through the MLST web interface. We demonstrate the versatility of our approach by anayzing both real and synthesized data from MLST databases. The introduced method for semi-supervised classification of sets of query STs is freely available for Windows, Mac OS X and Linux operative systems in BAPS 5.4 software which is downloadable at http://web.abo.fi/fak/mnf/mate/jc/software/baps.html. The query functionality is also directly available for the *Staphylococcus aureus *database at http://www.mlst.net and shortly will be available for other species databases hosted at this web portal.

**Conclusions:**

We have introduced a model-based tool for automated semi-supervised classification of new pathogen samples that can be integrated into the web interface of the MLST databases. In particular, when combined with the existing metadata, the semi-supervised labeling may provide invaluable information for assessing the position of a new set of query strains in relation to the particular pathogen population represented by the curated database.

Such information will be useful both for clinical and basic research purposes.

## Background

The widespread availability of DNA sequencing technology over recent years has lead to the widely adopted practice of routinely characterizing bacterial samples in terms of molecular variation over a set of core genes that have been established by the international research community for the organism in question [1,2]. Given success of the technologies behind these community-based efforts, there are now Multi-Locus Sequence Typing (MLST) databases available for many bacterial species, most hosted at http://www.mlst.net and http://www.pubmlst.org. These provide access to a vast amount of information about many important pathogens. More recently, geographical tools such as Google Maps, have been integrated into the databases for quick access and visualization of spatial data related to strain distribution. For examples of these advances, see http://www.spatialepidemiology.net/ and http://maps.mlst.net/. Another example of the evolution of these tools is the portal http://www.emlsa.net/, which provides access to electronic taxonomy of bacteria, through a common format and software for assigning strains to species via the Internet. Nevertheless, there is still substantial potential for global advances in pathogen epidemiology as the community using these tools keeps increasing and new functionality will be added on a continuous basis.

Thus far, the MLST information content displayed through the web access to either spatial or non-spatial data is based on relatively light procedures when considered from a statistical and/or computational perspective. This is reasonable, since the majority of more advanced model-based statistical methods for analyzing such data would not be scalable to provide real-time online access to results for users. However, provided that a statistical method for analyzing the MLST data meets the requirement of reasonable scalability, it may become a highly useful epidemiological tool and gain popularity very rapidly within this research community. The eBURST program available at http://eburst.mlst.net/ is an example of such a success story [3], making evolutionary snapshots of relatedness among sampled strains of pathogens.

Currently, MLST databases can be queried in various ways, including comparison of DNA sequences for a new set of samples with those previously existing in the database. However, when samples contain strains not currently present in the curated database, a user does not have an automated access to information which enables assessment of the relation of these samples to the earlier detected evolutionary groups. Such information is useful for various epidemiological and clinical purposes, in particular when considering the virulence and resistance characteristics of the strains. To enhance the querying features of the databases, we introduce here a statistical method for providing rapid access to probabilistic assignment of new strains to either pre-detected or earlier unseen evolutionary groups. The method is based on extending a Bayesian unsupervised classification method for MLST data [4] to a semi-supervised setting, where the existing curated MLST database plays the role of training data. To be able to handle the computational challenge of doing inference for the semi-supervised classification model, we adopt the computational strategies based on a stochastic optimization algorithm for unsupervised classification which are implemented in the BAPS software [5-7]. In contrast to more conventional Markov chain Monte Carlo (MCMC) methods for Bayesian inference, our algorithm is able to handle the computational issues more efficiently, such that the method can be applied to online use for MLST database queries. Alternative computationally fast approaches could also be developed by considering some of the recent advances in methodology for the analysis of genetic population structure [8,9], based on principal component and discriminant analysis.

## Methods

### Bayesian semi-supervised classification model

Standard MLST databases contain DNA sequences for 7 housekeeping genes shared by a pathogen species or a species group. Typical lengths of these genes vary in the range 350-500 basepairs. Let *g *= 1, ..., 7, denote the index of a single MLST gene and **x***_ig _*the observed DNA sequence for gene *g *in strain *i*. It is assumed that the sequences **x***_ig _*are aligned and of the same length *d_g _*for all considered strains. The total set of sequences for each strain is written as **x***_i_*. Each element *x_ijg _*in **x***_ig _*belongs to the finite alphabet , which is uniquely mapped to a set of integers such that we get the sample space  for each site *j*, *j *= 1, ..., *d_g_*. However, to obtain a less parameter-heavy classification model, we define the sample spaces for all 3-mers in these sequences in a more parsimonious manner (for details see below).

Corander and Tang [4] introduced a Bayesian second-order Markov model for unsupervised classification of MLST sequence data, which aims at a balance between a parsimonious parametrization and an adequate representation of dependencies in observed nucleotide frequencies among neighboring sites. Such standard Markovian structures are ubiquitous in statistical modeling of DNA sequences. Here we adapt this modeling framework to a semi-supervised setting, where training data are used to pre-specify a finite set of *k*_1 _possible distinct sources of new test strains, while not excluding the possibility that some (or even all) of these have emerged from a previously unseen evolutionary group. Let *V_g _*= {1, ..., *d_g_*} denote the index set of the site variables *x_ijg _*and *G_g _*= *G_g_*(*V_g_*, *E_g_*) an undirected graph on the node set *V_g _*with the edges in set *E_g_*. The edge set is determined by a second-order Markov structure where for any pair {*j*, j*} of site indices {*j*, j*} ∈ *E_g _*if and only if |*j *- j*| *<*3. Given the standard properties of decomposable graphical models [10], such a dependence structure leads to an explicit factorization of the joint probability distribution of site patterns given a joint classification of the training and query data. To define the factorization we let *cl*(*G_g_*) and *sep*(*G_g_*) denote the sets of cliques and the set of separators of the cliques of graph *G_g_*, respectively. The cliques correspond to all the triplets of consecutive site indices, whereas the separators correspond to all the pairs of consecutive site indices, except the first and last pairs for each gene.

Assuming there are in total *n *strains in a particular query, we index them by the set of integers *N *= {1, ..., *n*}. The observed sequence data for any subset *s *⊆ *N *of query strains is given by the collection **x**^(*s*) ^= {**x***_i _*: *i *∈ *s*}, and hence **x**^(*N*) ^represents the entire query data set. The sequence types (STs) existing in a curated MLST database are used as labeled training data, indexed by *M *= {1, ..., *m*}. The labels are assumed to be specified by an earlier analysis of the database contents, which divides the *m *STs into *k*_1 _distinct evolutionary groups using, for instance, an unsupervised classification with the BAPS software. The labeling *T *of the training data is a joint classification of the *m *STs into *k*_1 _classes and we use **z***_ig_*, **z***_i_*, **z**^(*s*)^, **z**^(*M*) ^for training data in a notation analogous to the query data as defined above.

For a set *a_g _*of sequence sites indexed by *V_g_*, such that the cardinality |*a_g_*| equals three, we let **x***_iag_*, **z***_iag _*be the corresponding 3-mers observed in gene *g *for strain *i *in the query and training data sets. Further, we let  equal the total number of distinct 3-mers observed at the sites *a_g _*in the joint collection of query and training data: .

Let *S *denote a joint classification of the *n *query STs into the *k*_1 _≥ 1 sources labeled by training data and *k*_2 _≥ 0 putative novel sources. Thus,  defines a (possibly) partially labeled partition of *N *(semi-supervised classification), such that  and *s_c _*∩ *s_c' _*= ∅, for all pairs {*c*, *c'*} ranging between 1, ..., *k*_1 _+ *k*_2_. The partition is completely labeled (supervised classification) when *k*_2 _= 0, that is when no query STs are assigned into previously unknown sources. We use  to denote the space of possible values of the semi-supervised classification structure *S*, conditional on a user-specified upper bound for *k*_1 _+ *k*_2_.

The joint conditional likelihood of query data given the classification *S *and the training data labeling *T *is under our Markov model defined as(1)

where *b_g _*and  are defined for subsets in *sep*(*G_g_*) analogously to the subsets in *cl*(*G_g_*), ,  are the probabilities of observing the *l*th 3-mer and 2-mer, respectively, in class *c*, and ,  are sufficient statistics corresponding to the observed counts of the *l*th 3-mer and 2-mer in class *c*. Parameter *θ *in (1) is used as a joint abbreviation for all the continuous parameters in the expression, which correspond to probabilities of observing the particular site patterns within the classes. Notice that the probabilities  and counts  are unambiguously determined by marginalization from  and , since each *b_g _*is a subsequence of a *a_g _*with cardinality equal to two, which follows from the order of the Markov model. Since the probabilities  are unknown parameters, the training data are used for learning them for the *k*_1 _*a priori *known classes, whereas only non-informative prior distributions are used for inferences about the remaining *k*_2 _classes. Furthermore, since these probabilities are nuisance parameters regarding the classification task, they should be integrated out when making inferences about the classification *S*.

Assuming standard Dirichlet  prior distributions which are factorized with respect to the graphs *Gg *for all components of *θ *[10,11],we can derive an analytical expression for the posterior probability *p*(*S*|**z**^(*M*)^, **x**^(*N*)^, *T*) of *S*. The conjugate Dirichlet prior is widely adopted in particular in bioinformatics applications due to the computational advantage provided by analytical marginalization over frequency (nuisance) parameters in multinomial models. The posterior of *S *equals(2)

where *p*(*S*|*T*) *>*0 is the prior probability of *S *and *f*(**z**^(*M*)^, **x**^(*N*)^, *T*) is a normalizing constant equal to the sum(3)

In the expressions below  is the observed count of the *l*th 3-mer from the training data on class *c *and is the corresponding marginalized count. The first one of the two above integrals can be written in detail as(4)

where the term  equals(5)

which further simplifies to the expression(6)

where . Correspondingly,  equals(7)

which in turn simplifies to(8)

where .We set all the Dirichlet hyperparameters in  equal to the reference value , which is generalization of the Jeffreys' prior and reflects *a priori *symmetry with respect to the 3-mer values. For a detailed discussion about such reference priors see [11]. The prior distribution of *S *is set equal to the uniform distribution in , which is defined as(9)

where  refers to the cardinality of the space . Similarly, the second integral can be written as(10)

where again(11)

and(12)

since the previously unseen sources lack the training data observations.

We define our joint semi-supervised classifier as the classification structure *Ŝ *corresponding to the posterior mode over the distribution specified in (2)(13)

Given *Ŝ*, one may calculate the conditional posterior distribution over possible assignments of the *n *query STs according to(14)

where *Ŝ*(*i *∈ *s_c_*) is the mode classification with *i*th query strain re-assigned to class *c*. These probabilities reflect the local posterior uncertainty about the possible sources of the query STs and they can be calculated in a simple manner using the above analytical expressions. In the next section it is shown how fast stochastic optimization can be used to obtain a plausible estimate of *Ŝ *in the online setting considered here.

### Inference algorithm

A standard Bayesian supervised classifier, for example the naive Bayes classifier [12], would treat each query ST separately and assign it to the class maximizing the posterior probability among the *k*_1 _known alternative sources. Such an approach has very modest computational complexity and it can be easily extended to the semi-supervised classification task where any single query ST is allowed to be assigned to an additional class lacking training data. However, considering the query STs individually has the disadvantage that when multiple STs are assigned to a previously unknown evolutionary group, the classifier provides no information about whether they should be interpreted as a single group or eventually be split into multiple novel lineages. In addition, when compared to a simultaneous classification, separate classification of all query STs offers lower statical power to detect strains from novel groups which are only modestly distinct from the *k*_1 _groups in the training data. On the other hand, simultaneous semi-supervised classification of the query STs is computationally substantially more challenging than a separate classification, since the search operators must allow for the presence of multiple novel subsets of strains. Standard Bayesian computational tools, such as the Gibbs sampler [13], provide a straightforward way to implement a simultaneous semi-supervised classifier. However, due to their notoriously slow convergence for mixture models, they do not offer a highly versatile solution for an online application where query assignments are expected to be provided on a nearly real-time basis. Hanage et al. [14] analyzed a large MLST database for which they concluded that a Gibbs sampler based approach did not converge with a reasonable computational effort (~3 days on a single CPU). The same convergence issue was also explored for a different data type in [15], where Gibbs sampler and a stochastic greedy search algorithm were compared. Therefore, we use for semi-supervised classification the same efficient non-reversible stochastic search operators that are used for unsupervised classification of MLST data in the BAPS algorithm.

Given a set of query data and a preprocessed MLST database in which STs are divided into *k*_1 _groups, it is necessary to determine first the total number  of distinct 3-mers observed in the joint collection of query and training data for all collections of sites *a_g _*over the genes. This requires a linear scan of the observed sequences in the query data. Additionally, pairwise Hamming sequence distances are calculated for all pairs of query STs, as these are used to guide the stochastic search of the optimal classification. Notice that the unnormalized posterior probability distribution over the possible assignments *S *of query strains is uniquely determined by the sufficient statistics  and the Dirichlet prior hyperparameters . Therefore, an efficient algorithm for searching the classification maximizing the posterior can be efficiently constructed by book-keeping changes in the sufficient statistics implied by re-assignments of subsets of query strains. The search operators in  that are used for improving any current state *S *of the simultaneous assignment of query STs work as follows:

1. In a random order relocate each single ST to the class in *S *that leads to the maximal increase in the posterior probability (2). This operator considers explicitly the assignment of each ST into a new singleton class, unless that would increase the number of classes *k*_2 _beyond the user-specified upper bound.

2. Merge STs in the two classes of *S *which leads to the maximal increase in the posterior probability (2). If no putative merge increases the probability, the state of *S *is not altered. Notice that this operator applies to all classes irrespectively of their size, thus including any potential singleton classes introduced by the first or third operator.

3. In a random order, split each class into two maximally homogeneous subclasses using the complete linkage clustering algorithm with Hamming distances between the query STs. If a classification S* after split is associated with higher posterior probability than the current classification *S*, the split is accepted and otherwise it is rejected.

4. In a random order over the classes of *S*, simultaneous relocation of several STs from each class is attempted. The STs in a class are first sorted into a decreasing order with respect to the improvement in posterior probability (2) when they are assigned one-by-one into some other class, that is the ST associated with the largest improvement is placed first in the sorting etc. A candidate for new classification structure S* is then formed by relocating STs in this order to the class which leads to the largest increase in (2) or to the smallest decrease if no positive changes are possible. The relocation is continued either until the the total change in (2) becomes positive, in which case the candidate S* is set as the next state of the search algorithm, or until all STs in the class are relocated and the total change remains negative, in which case the candidate is rejected.

The search algorithm uses each of the above operators in varying combinations until no improvement in (2) is achievable after two consecutive attempts. Given its efficient implementation, even in an online application the algorithm can be independently run multiple times such that the globally best classification over the runs is chosen as the final estimate of the posterior mode classification. Multiple independent searches will reduce the probability that the best classification identified among them will be considerably suboptimal, representing a local peak in the posterior distribution. Since any two classification structures can be analytically compared, the searches can even be performed on separate processors and results later combined using the batch mode interface of the BAPS software.

## Results

We have implemented the semi-supervised classification algorithm for MLST data in the BAPS software version 5.4 which is available for Windows, Mac OS × and Linux operative systems. It can be accessed both through the graphical user interface or the batch mode interface, which simplifies automation of the use of the tool in MLST web interfaces. In this section we demonstrate the performance of the semi-supervised classification tool using data from two MLST databases. The first database http://pubmlst.org/bcereus/ is for the pathogen species *Bacillus cereus *[16] and the second database http://saureus.mlst.net/ is for the pathogen species *Staphylococcus aureus *[17].

We extracted multilocus DNA sequences for 515 and 1404 STs from the two databases, respectively. The housekeeping genes used in typing of the *B. cereus *are: *glpF *(glycerol uptake facilitator protein), *gmk *(guanylate kinase), *ilvD *(dihydroxy-acid dehydratase), *pta *(phosphate acetyltransferase), *pur *(phosphoribosylaminoimidazolecarboxamide), *pycA *(pyruvate carboxylase), *tpi *(triosephosphate isomerase). The housekeeping genes used in typing of the *S. aureus *are *arc *(Carbamate kinase), *aro *(Shikimate dehydrogenase), *glp *(Glycerol kinase), *gmk*, *pta*, *tpi*, *yqi *(Acetyle coenzyme A acetyltransferase). The lengths of the MLST loci for *B. cereus *vary between 348-504 basepairs and the total concatenated length of the sequences equals 2829 basepairs. For *S. aureus *the lengths vary between 402-516 basepairs, the total concatenated length being 3198 basepairs.

A number of simulation experiments were performed using the real *B. cereus *and *S. aureus *data as the basis. Firstly, we divided the two databases into distinct groups of STs using an unsupervised classification (clustering) analysis option available in BAPS software for MLST type data. This resulted in 11 and 6 groups for the *B. cereus *and *S. aureus *data, respectively. For *B. cereus *the group sizes varied between 9-127 STs and for *S. aureus *between 9-444 STs.

In the first experiment we chose randomly 30% of the database STs as query data and the remaining 70% were used as training data. The training data were pre-classified into the groups identified by the earlier unsupervised analysis and the query data were analyzed assuming that there are at most 10 novel groups present in it. This setup was replicated 10 times and we calculated for each random data configuration how well the labels of the query STs matched the pre-classification labels using the adjusted Rand Index (ARI) [18]. The average ARI over the replicates (with std.dev. in parenthesis) is 1.000 (0.000) and 0.999 (0.003) for the *B. cereus *and *S. aureus *data, respectively.

In the second experiment the database STs were not randomly chosen into the query data as such, but we selected instead randomly 30% of the database ST groups as query data (3/11 and 2/6 groups), while leaving the remaining groups as training data. Notice that in the first experiment every class that was previously identified from the database had approximately 30% of its STs included in the test data, and thus, the same underlying classes were present both in the training and test data sets. In contrast, in the second experiment the test data consisted of groups of STs which did not correspond to any groups present in the training data, and thus, the training and test data sets were completely non-overlapping in terms of underlying groups.

**Figure 1:**
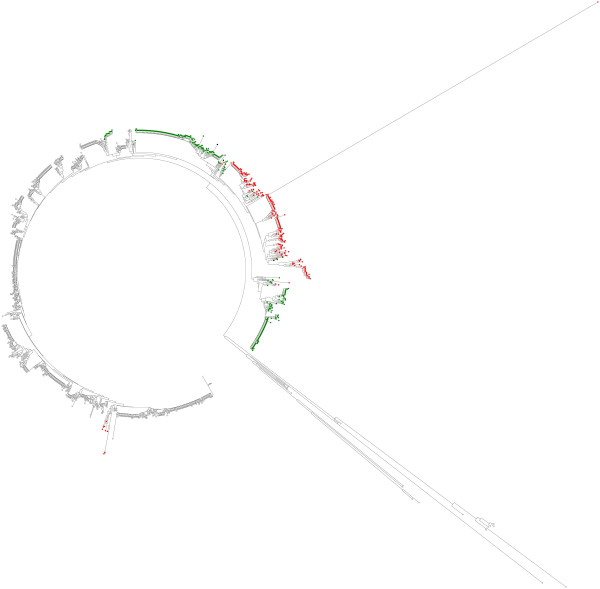
**Example of a semi-supervised classification of query STs from S. aureus database in the second experiment based on an annotated NJ tree**. The STs marked with red and green colors represent the query STs labeled as the two new detected groups and the uncolored STs represent the remaining training data groups.

The corresponding ARI values as in the first experiment are now 0.988 (0.029) and 0.966 (0.045) for *B. cereus *and *S. aureus *databases, respectively. To illustrate the data in the simulation experiments we made two Neighbor-Joining (NJ) trees annotated with pre-classification and novel labels. The trees were created with MEGA 4 software [19] using the maximum composite likelihood option. In Figure 1, the semi-supervised labeling is shown for one of the *S. aureus *database replicates in the second experiment. Here there are two novel groups of STs in the test data and only a single ST in one of them is mislabeled (uncolored in Figure 1) in the semi-supervised analysis. Note that BAPS groups may occasionally deviate from the groups derived from a phylogenetic tree, primarily due to presence of recombinant alleles in the data. For instance, all the long branches present in Figure 1 are due to a strongly deviating allele at a single locus, or even at two loci for some STs. We detected these cases by using the BRAT software [20] to screen the entire database (exact results not shown). When the deviating alleles were removed, all the long branches present in Figure 1 vanished, such that the corresponding STs closely resemble strains present in the remaining lineages. For instance, the single red-labeled ST with a very long branch had at one locus an allele with closest match to another species (*S. epidermidis*) when its DNA sequence was queried at the NCBI nucleotide collection, which could represent either a result of genuine inter-species recombination or a case of DNA contamination in the laboratory. Since BAPS recognized that the ST in question had very close resemblance to other red-labeled STs at the remaining six loci, the probabilistic query did not yield a label indicating separate origin.

It should also be noted that a small number of STs labeled as red reside in the NJ tree among green labeled taxa and conversely, a small number of STs labeled as green reside among red labeled taxa. Such a deviance has several possible explanations. Firstly, the labeling of these strains by the population genetic assignment may be erroneous, such that the tree correctly displays their origin. Secondly, due to the small evolutionary distances among these groups of strains, the NJ tree itself may provide a distorted view of their origin. In particular, under limited molecular resolution, the population genetic approach gains in a relative sense more statistical power to correctly detect lineage boundaries from a large sample in the presence of a small number of sites with highly characteristic nucleotides for a particular lineage, compared to a tree-based approach. This is primarily because the population genetic model directly compares nucleotide frequencies at sites within and between putative pools of samples and aims at answering a considerably simpler statistical question than a tree-based approach. We have obtained additional support for this tendency by examining data for *Burkholderia pseudomallei *STs, for which a large number of additional loci were available (exact data and results not shown). In general, in our bootstrap experiments BAPS assigned strains significantly to the correct lineage with a considerably smaller number of loci compared with a tree-based approach.

**Figure 2:**
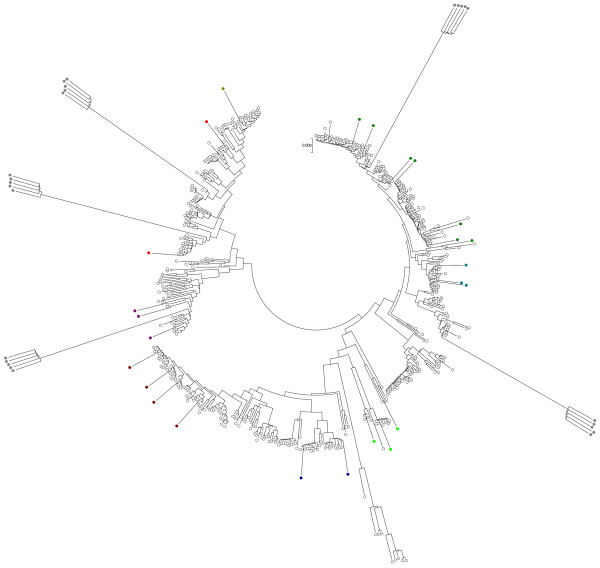
**Example of a semi-supervised classification of query STs from B. cereus database in the third experiment based on an annotated NJ tree**. The STs marked with grey colors are the new detected groups. The uncolored STs represent the STs in training data groups and the remaining colored STs are the 25 query STs that were correctly labeled by their respective groups.

In the third experiment we chose the *B. cereus *database and introduced random mutations in the sequences of the query STs. Two types of query STs were generated to mimic a situation where some new strains represent previously detected lineages, whereas the others are sampled from multiple unseen lineages. To create novel strains representing the first scenario, we chose randomly 25 STs from the database and introduced 1% of random mutations into their sequences. In addition, to create strains corresponding to the latter scenario, we randomly sampled 5 STs from the database and introduced 5% of random mutations to their sequences. Thereafter, 5 independent test strains were generated from each of these mutated STs by introducing further 1% of mutations to the sequences. The test data thus contains 50 query STs in total. Figure 2 illustrates the semi-supervised labeling of these data by showing simultaneously the training and test samples in an NJ tree. All 25 test STs representing previously sampled lineages, as well as all the five groups of STs from previously unsampled lineages were correctly labeled according to the group they were generated from.

The final experiment was performed to investigate the computational cost of applying our method to an online probabilistic query for an MLST database. We chose the following four sizes of query sets of STs to represent a wide range of typically expected queries: 5, 10, 50 and 100 STs. In each replicate of the experiment, the indicated number of STs were randomly chosen as test data and excluded from the database, while the remaining STs were used as training data. Independent point mutations were introduced to the sequences of test STs before submitting them as a query, such that on average nucleotide values at 1% of the sites were changed for the *B. cereus *STs and at 0.5% of the sites for the *S. aureus *STs. In total 10 replicates were performed on a PC with a 2.66 GHz processor and the mean time in seconds (SD within parenthesis) from the query submission to the final estimates of posterior assignment probabilities was for *B. cereus*: 0.320 (0.091), 3.887 (0.596), 56.175 (13.967), 181.705 (18.294), for the four distinct query set sizes, respectively. For *S. aureus *the corresponding computation times were: 0.920 (0.034), 10.945 (2.102), 95.812 (12.966), 334.339 (84.523). These results illustrate that our method can easily be applied in an online query setting, as the required computation time is at most a couple of minutes even for large query sets. It is also worth noticing that the query sets are not expected to be that large in a majority of cases within clinical applications of MLST.

## Discussion

The epidemiological research community has with its combined efforts enabled a major leap forward in the understanding of the dynamics and evolution of major human and animal pathogens through the MLST web software. As all the MLST databases are continuously increasing in size and the popularity of these typing schemes continues to grow, the need of additional tools for rapidly simultaneously interfacing both previously curated and new data has emerged as well. Our example experiments based on real MLST databases illustrate that the model-based approach provides high accuracy in correctly labeling both strains from groups existing in the curated database as well as strains representing previously unseen lineages. In addition, our method provides a probabilistic characterization of the assignment uncertainty in terms of posterior probabilities calculated over the possible putative sources in the estimated mode classification structure. A classification framework where each query ST is labeled independently of other strains would provide a much simpler solution to the assignment problem in computational terms, however, on the other hand it is a more statistically coherent approach to handle all the query strains within a joint modeling framework to increase statistical power to detect samples from previously unseen evolutionary groups. It is worth noticing that since there is no other probability-based method available that would be tailored to MLST type data, we have not considered the semi-supervised classification task in a comparative fashion.

## Conclusions

We have introduced a model-based tool for automated semi-supervised classification of new pathogen samples that can be integrated into the web interface of the MLST databases. In particular, when combined with the existing metadata, the semi-supervised labeling may provide invaluable information for assessing the position of a new set of query strains in relation to the particular pathogen population represented by the curated database. Such information will be useful both for clinical and basic research purposes.

## Competing interests

The authors declare that they have no competing interests.

## Authors' contributions

TC introduced the original idea of using a model-based approach to semi-supervised classification of novel MLST sequence types. LC and JC designed and implemented the classification model, the stochastic inference algorithm and the computational experiments. TC and DMA developed the interface to MLST databases. BGS provided expertise on the clinical importance and use of MLST database information. All authors contributed to writing of the manuscript. All authors read and approved the final manuscript.
